# Psychiatric and Cognitive Effects of Testosterone Therapy in Adult Men: A Systematic Review of Clinical Evidence and Mechanistic Insights

**DOI:** 10.7759/cureus.102894

**Published:** 2026-02-03

**Authors:** Luis M Canal de Velasco, José Emiliano González Flores, Laura Vargas, Jose Luis Morales Arteaga

**Affiliations:** 1 Medical Education and Simulation, Panamerican University, Mexico City, MEX; 2 Surgery, Tecnológico de Monterrey Campus Ciudad de Mexico, Mexico City, MEX; 3 School of Medicine, University of Colorado, Anschutz Medical Campus, Aurora, USA; 4 Cardiology, Panamerican University, Mexico City, MEX

**Keywords:** cognition, depression, hypogonadism, memory, mood, neuroendocrinology, psychiatric outcomes, randomized controlled trial, testosterone therapy, visuospatial function

## Abstract

Testosterone plays a critical role in male physical and psychological health, influencing not only reproductive and metabolic functions but also mood, cognition, and overall mental well-being. Age-related testosterone decline has been associated with depressive symptoms, cognitive impairment, and reduced quality of life. Although testosterone therapy (TT) is well established for somatic outcomes, its psychiatric and cognitive effects remain inconsistently characterized across clinical trials.

The objective is to systematically review the evidence on the effects of TT on psychiatric and cognitive outcomes in adult men, compared with placebo or standard care, and to evaluate associated safety findings. This systematic review followed PRISMA guidelines and was registered in PROSPERO (ID: 1163108). Comprehensive searches were conducted in MEDLINE, EMBASE, Cochrane Library, ScienceDirect, and Google Scholar up to October 2025. Eligible studies included randomized controlled trials (RCTs) and observational studies evaluating TT (any route or dose) in adult men with psychiatric or cognitive endpoints. Data extraction was performed independently by two reviewers, using the PICOS framework. Risk of bias was assessed using the Cochrane RoB 2 tool.

A total of 11 RCTs, encompassing over 600 male participants (aged 18-85 years), were included. Populations ranged from healthy eugonadal men to older hypogonadal adults, and patients with treatment-resistant depression (TRD) or mild Alzheimer’s disease. TT significantly improved depressive symptoms in men with TRD (p < 0.05), and enhanced specific cognitive domains - particularly verbal memory and visuospatial processing (p < 0.05) - in older or hypogonadal men. Global cognition and anxiety outcomes showed inconsistent effects. Quality of life and sexual function consistently improved across studies, while adverse events were mild and transient (mainly acne, edema, or skin irritation). No major cardiovascular, hepatic, or thromboembolic complications were reported.

However, evidence certainty remains moderate due to small sample sizes, brief intervention periods, and heterogeneous methodologies. TT should be considered a complementary, not primary, approach in the management of depressive and cognitive symptoms in hypogonadal men, implemented under endocrinological supervision, with regular biochemical and clinical monitoring. Larger, long-term RCTs are warranted to confirm efficacy, optimize dosing, and define long-term neuropsychiatric safety.

## Introduction and background

Testosterone, the principal androgen in men, plays a fundamental role not only in reproductive and physical health, but also in brain function and psychological well-being. Age-related testosterone decline affects a substantial proportion of aging men and has been consistently associated with depressive symptoms. It is frequently observed among men with depressive symptoms, underscoring its clinical relevance beyond reproductive health. Several epidemiological and clinical studies suggest that androgen deficiency becomes increasingly prevalent with advancing age, and is frequently observed among men with treatment-resistant depression (TRD), highlighting its clinical relevance beyond reproductive health [1-5].

Beyond its well-established influence on sexual function, muscle mass, bone density, and metabolic regulation, growing evidence indicates that testosterone meaningfully affects mood regulation, cognition, motivation, and overall mental health, through androgen receptors widely distributed across limbic and prefrontal circuits [1,2]. These neurobiological pathways - spanning synaptic plasticity, neurotransmitter modulation, neurogenesis, and neuroinflammatory control - provide a plausible mechanistic basis for testosterone’s observed effects on mood regulation, motivational drive, cognitive processing, and resilience to neuropsychiatric disorders in hypogonadal men [3,4]. Despite growing evidence in the literature on testosterone’s benefits for mood and cognition in hypogonadal and older men, a comprehensive summary and critical review of the existing findings has not been conducted. This study addresses this gap by systematically evaluating current evidence on testosterone treatment and its effects on mood and cognition, thereby providing an updated framework that advances understanding and guides future clinical research in this area.

Age-related androgen decline and clinical hypogonadism are associated with depressive symptoms, reduced motivation, cognitive slowing, and diminished quality of life; in parallel, clinical use of testosterone therapy (TT) has expanded to address these domains alongside somatic outcomes [2]. Contemporary reviews and trials suggest that, when appropriately prescribed and monitored, TT improves sexual function, lean body mass, bone mineral density, insulin sensitivity, vitality, and select aspects of neuropsychological health [3]. Importantly for this review’s focus, randomized controlled trials (RCTs) and observational studies indicate that TT can alleviate depressive symptoms and may enhance specific cognitive domains - particularly memory and executive processes - though results vary by population, baseline androgen status, dose, formulation, and follow-up duration [4-6]. This heterogeneity, together with inconsistent outcome measures and short study horizons, complicates inference and underscores the need for an integrative synthesis centered on psychiatric and cognitive endpoints. Despite numerous clinical trials, heterogeneity in patient populations, androgen formulations, dosing regimens, and outcome measures has produced inconsistent findings. Moreover, prior reviews have often focused on metabolic or sexual outcomes, leaving psychiatric and cognitive endpoints underexplored. This gap highlights the need for an updated, integrative synthesis of clinical evidence.

The present systematic review, therefore, addresses the central question: How does TT influence psychiatric and cognitive outcomes in adult men, compared with placebo or standard care? Existing evidence suggests that TT is associated with measurable improvements in mood, reductions in depressive and anxiety symptoms, and enhancements in cognitive performance - particularly in memory, attention, and executive function. By synthesizing and critically appraising clinical evidence across adult male populations - including age-stratified perspectives from men in their 40s and those aged ≥50 years - this review aims to clarify therapeutic effects and safety signals relevant to mental health and cognition, and to inform evidence-based, patient-centered decision-making in androgen therapy [1,2]. 

## Review

Methods and materials

Literature Search Strategy

This systematic review was conducted in accordance with PRISMA guidelines and followed the protocol registered in the International Prospective Register of Systematic Reviews (PROSPERO; ID: 1163108). A comprehensive literature search was performed in September 2025 and updated in October 2025 across the databases MEDLINE (via PubMed), EMBASE, Cochrane Library, Google Scholar, and ScienceDirect, without restriction by publication date and limited to studies published in English.

The search strategy was designed to capture all clinical studies examining the psychiatric and cognitive effects of TT in adult male populations. The following combination of keywords and MeSH terms was applied using Boolean operators: (“testosterone” OR “androgen replacement” OR “testosterone therapy” OR “testosterone supplementation” OR “testosterone gel” OR “testosterone injection”) AND (“depression” OR “anxiety” OR “mood” OR “aggression” OR “cognition” OR “memory” OR “executive function” OR “verbal memory” OR “spatial memory” OR “Alzheimer’s disease” OR “cognitive decline”). Reference lists of included studies and relevant reviews were also screened manually to identify additional eligible articles.

Methodology for Selecting Studies

Two reviewers (JE and EM) independently screened the titles and abstracts of all identified articles to determine eligibility for inclusion in this systematic review. Articles were retrieved from the databases described above and screened using the Rayyan platform (Qatar Computing Research Institute, Ar-Rayyan, Qatar; https://www.rayyan.ai/) [7]. When titles and abstracts lacked sufficient detail for an inclusion decision, the full-text articles were retrieved and assessed. Studies were included if they met the following criteria: the eligibility criteria were defined according to the PICOS framework. The Population (P) included adult male participants 18 years or older. The Intervention (I) comprised TT administered via any route, including transdermal, intramuscular, or oral formulations. The Comparison (C) groups consisted of placebo, standard care, or pre/post-comparisons in observational designs. The Outcomes (O) of interest were psychiatric measures - such as mood, depression, anxiety, aggression, and quality of life - and cognitive domains, including memory, attention, and executive function. Finally, the Study Design (S) encompassed RCTs, prospective or retrospective cohort studies, cross-sectional investigations, case series, and case reports published in English. Exclusion criteria included: (1) non-testosterone interventions, (2) studies without psychiatric or cognitive outcomes, (3) preclinical or in vitro studies, (4) reviews, editorials, or opinion papers, (5) non-English publications, and (6) duplicate or overlapping datasets.

Any disagreements between the two reviewers during the selection process were resolved through discussion and consensus, and, when necessary, by consulting a third independent reviewer (JLC).

Process for Data Extraction

Data extraction was conducted independently and in duplicate using a predefined, standardized form in Microsoft Excel (Microsoft® Corp., Redmond, WA, USA) that included publication year, study design, population characteristics, intervention details, psychiatric and cognitive outcomes, adverse events, follow-up, and reported effect sizes. When data were missing or unclear, corresponding authors were contacted for clarification.

In addition, data on psychiatric and cognitive outcomes included assessment tools used (e.g., Hamilton Depression Rating Scale (HAM-D), Beck Depression Inventory (BDI), Hamilton Anxiety Rating Scale (HAM-A), Alzheimer’s Disease Assessment Scale - Cognitive Subscale (ADAS-Cog), Mini-Mental State Examination (MMSE)), baseline and post-intervention scores, changes in mood, depression, anxiety, aggression, cognition, memory, and quality of life. Other extracted parameters included adverse events, treatment-related complications, follow-up duration, study strengths and limitations, clinical recommendations, significant outcomes, reported effect sizes, and levels of evidence according to the Oxford Centre for Evidence-Based Medicine (OCEBM) classification. Any discrepancies in data extraction were resolved through discussion and consensus, and, if necessary, by consultation with a third independent reviewer.

Due to significant heterogeneity in study design, intervention types, outcome measures, and reporting formats, a quantitative meta-analysis was not feasible. Therefore, data were synthesized qualitatively following a narrative synthesis approach, emphasizing directionality and consistency of effects across studies.

Assessment of Quality and Bias Risk

We evaluated risk of bias for all included RCTs using the Revised Cochrane Risk-of-Bias Tool for Randomized Trials (RoB 2). JE and EM independently assessed studies across five standard domains: (1) bias arising from the randomization process, (2) bias due to deviations from intended interventions, (3) bias due to missing outcome data, (4) bias in the measurement of the outcome, and (5) bias in the selection of the reported result. For crossover trials, an additional domain assessing potential carry-over effects was also considered. Any disagreements were resolved by consensus or by a third reviewer.

Results

**Figure 1:**
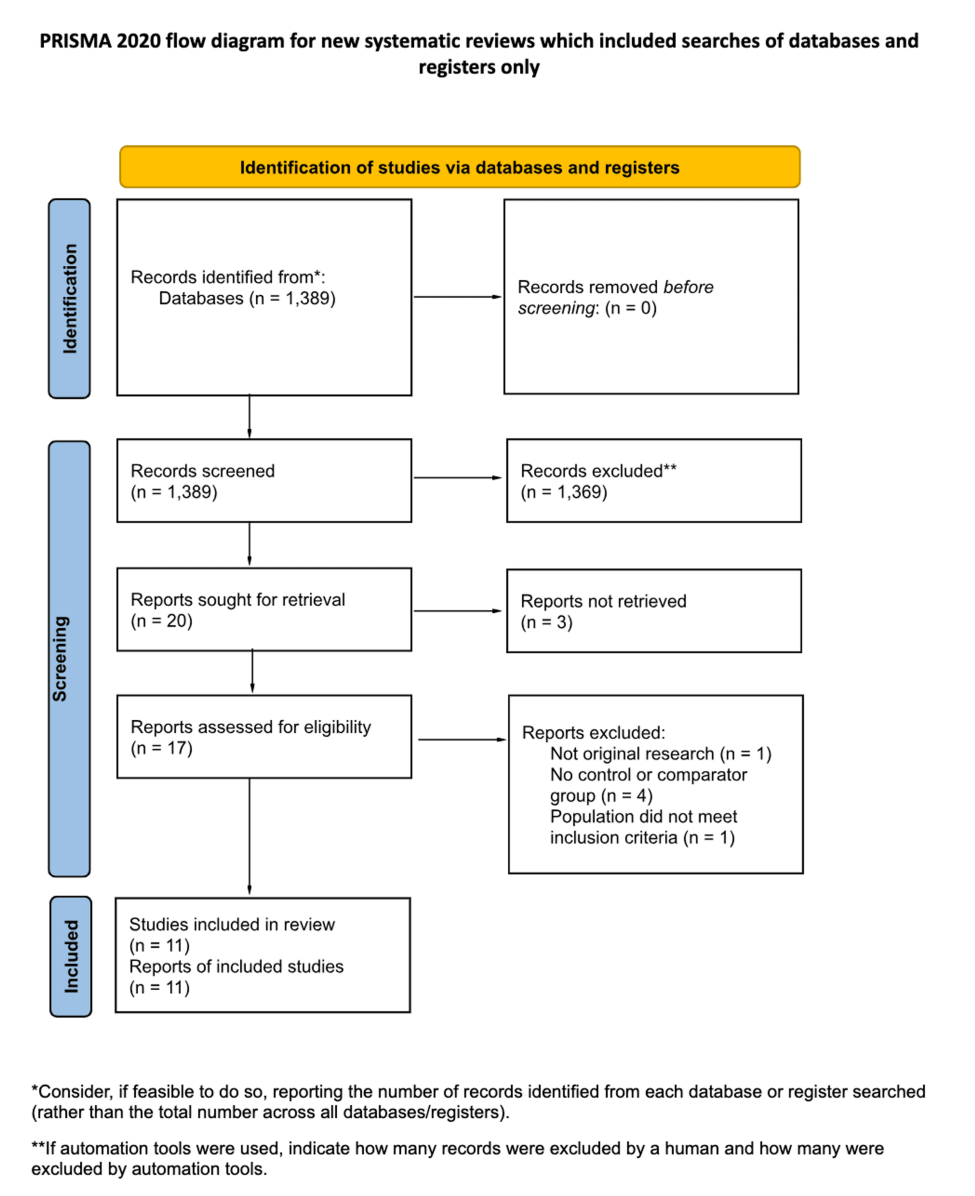
Flow diagram summarizes the systematic screening process following PRISMA 2020 guidelines. A total of 1,389 records were identified from databases, with none from registers. After initial screening, 1,369 records were excluded. Among 20 reports sought for retrieval, three could not be accessed. From the 17 full-text studies assessed for eligibility, six were excluded: one was not original research, four lacked a control group, and one involved a non-eligible population. Eleven studies fulfilled all inclusion criteria and were incorporated into the systematic review.

Although the search strategy included observational and non-RCT studies, no studies with these designs met the inclusion criteria. Therefore, only 11 RCT studies were included in the final list of included studies (after undergoing full-text screening), which were published between 1997 and 2015 (see PRISMA flow diagram, Figure 1). The initial database search identified 1,389 records, of which 20 full-text articles were assessed for eligibility and 11 met all inclusion criteria.

Depression and Mood Outcomes

TT demonstrated significant antidepressant effects, particularly as an adjunctive treatment in men with TRD. In RCTs, adjunctive testosterone led to marked reductions in HAM-D scores compared with placebo (p < 0.05) [8,9]. In contrast, monotherapy trials in hypogonadal men with major depressive disorder (MDD) showed mixed results, likely due to high placebo response rates [10]. Among young eugonadal men, supraphysiological testosterone doses modestly increased aggression (p = 0.04) but had no significant effect on overall mood [11].

Cognitive Outcomes

Moderate testosterone supplementation enhanced domain-specific cognition, particularly verbal memory and visuospatial processing, with dose-dependent effects observed in healthy older men and hypogonadal populations [12,13]. In contrast, in men with mild Alzheimer’s disease, TT was associated with modest improvements in global cognitive measures, such as ADAS-Cog and MMSE, without consistent evidence of domain-specific or dose-related cognitive enhancement [14,15].

Quality of Life and Functional Outcomes

TT consistently improved quality of life and sexual function. Steidle et al. [16] reported a 35% rise in sexual activity and significant gains in libido (p < 0.01), while Lu et al. [17] observed a 23% improvement in caregiver-rated quality of life among Alzheimer’s patients.

Complications and Safety

Across all trials (n = 600), TT was well tolerated, with no major cardiovascular, thromboembolic, or hepatic events reported. Minor effects such as mild edema (<5%), injection-site reactions (<4%), and acne (<3%) were transient and self-limiting. Hematocrit increases were reported in some studies and occasionally required discontinuation or intervention [13,15,18]. 

Risk of Bias and Evidence Quality

Methodological quality was generally high. Most RCTs showed low-to-moderate risk of bias, with minor concerns related to attrition, incomplete data reporting, and potential carry-over in crossover designs. Randomization and blinding procedures were well described, and validated outcome measures were used across studies. Evidence quality was strongest for antidepressant and domain-specific cognitive effects (Level I), while evidence for global cognition and long-term psychiatric outcomes remained moderate (Level II).

Overall, TT exerts measurable antidepressant and cognitive-enhancing effects, especially as adjunctive treatment in TRD and as a cognitive enhancer in older hypogonadal men. The cumulative evidence corresponds to Level I-II quality. While short-term safety is well established, long-term neuropsychiatric and dose-response effects require further investigation.

The main characteristics of the included studies, including sample size, population characteristics, intervention type, duration, and strengths, weaknesses, or clinical recommendations, are summarized in Tables 1-2. Collectively, these studies encompassed over 600 male participants aged 18 to 85 years. Study populations varied widely, including healthy young men, older hypogonadal men, patients with mild Alzheimer’s disease, and individuals with TRD. Most trials were double-blind, randomized, and placebo-controlled, with treatment durations ranging from 6 weeks to 12 months, and are summarized in Table 3.

**Table 1:** Characteristics of included randomized controlled trials evaluating testosterone therapy and neuropsychiatric or cognitive outcomes in adult men. Abbreviations: RCT, randomized controlled trial; TT, testosterone therapy; IM, intramuscular; GnRH, gonadotropin-releasing hormone; SSRI, selective serotonin reuptake inhibitor; wks, weeks; mo, months; y, years; q2w, every 2 weeks; q14-17d, every 14 to 17 days; T, serum testosterone; AA2500, androgen application formulation 2500 (testosterone gel); mg, milligrams; g, grams; day, per day; wks/phase + washout, each treatment phase followed by washout period; Placebo, inactive comparator matching the intervention vehicle.

Authors (years)	Study design	Country	Age range (years)	Population		Intervention vs comparator	Duration of intervention
				Sample size in intervention group (n)	Sample size in control group (n)		
Asih et al. (2015) [12]	RCT - Crossover	Australia	50-80 y	37	37	Transdermal TT (physiologic dose) vs placebo	12 wks/phase + washout
O’Connor et al. (2004) [11]	RCT - Double-blind - Crossover	United States	19-35 y	56	56	IM TT (physiologic dose) vs placebo	6 wks/phase + washout
Gray et al. (2005) [18]	RCT - Dose-ranging - Controlled	United States	60-75 y	60	60	Weekly IM testosterone enanthate (25-600 mg) + GnRH agonist vs placebo	20 wks
Seidman et al. (2005) [8]	RCT - Double-blind - Placebo-controlled	United States	30-65 y	15	15	IM testosterone cypionate 200 mg q2w as adjunct to SSRI vs placebo injection	6 wks
Lu et al. (2006) [17]	RCT - Double-blind - Placebo-controlled	United States	>50 y	16	16	1% transdermal TT gel vs placebo gel	24 wks
Steidle et al. (2003) [16]	RCT - Multicenter - Multidose - Active and placebo-controlled	United States	20-80 y	99	102	AA2500 TT gel (50-100 mg/day) vs TT patch (5 mg/day) and placebo gel	90 days
Cherrier et al. (2007) [13]	RCT - Double-blind - Placebo-controlled	United States	50-90 y	43	14	Weekly IM testosterone enanthate (50-300 mg) vs placebo injections	6 wks
Seidman et al. (2001) [10]	RCT - Double-blind - Placebo-controlled - Parallel-group	United States	35-71 y	13	17	IM testosterone enanthate 200 mg weekly vs placebo (sesame oil)	6 wks
Sih et al. (1997) [15]	RCT - Double-blind - Placebo-controlled	United States	51-79 y	17	15	IM testosterone cypionate 200 mg q14-17d vs placebo (saline)	12 mo
Tan and Pu (2003) [14]	RCT - Double-blind - Placebo-controlled - Pilot	United States	67-82 y	5	5	IM testosterone enanthate 200 mg q2w vs placebo	12 mo
Pope et al. (2003) [9]	RCT - Double-blind - Placebo-controlled	United States	30-65 y	12	10	1% testosterone gel 10 g/day (reduce to 7.5 g if T >1070 ng/dL) vs placebo gel	8 wks

**Table 2:** Summary of key findings, methodological quality, and clinical implications of included randomized controlled trials. Levels of evidence (adapted from the Oxford Centre for Evidence-Based Medicine): Level I: High-quality RCTs with a low risk of bias or consistent findings across multiple RCTs; Level II: Moderate-quality RCTs or small-sample RCTs with limitations in duration, power, or generalizability; Level III: Pilot or preliminary RCTs, open-label studies, or nonrandomized controlled trials with limited external validity. Note: p-values are reported as presented in each study. “NS” denotes nonsignificant results (p > 0.05). Cognitive outcomes include domain-specific measures (e.g., verbal, visuospatial, or executive functions) rather than global cognition, unless otherwise specified. Abbreviations: ADAS-Cog, Alzheimer’s Disease Assessment Scale-Cognitive Subscale; BDI, Beck Depression Inventory; CDT, Clock Drawing Test; CGI, Clinical Global Impression; DSPS-M, Derogatis Sexual Functioning Scale-Male Version; DHT, dihydrotestosterone; HAM-D, Hamilton Depression Rating Scale; IM, intramuscular; MMSE, Mini-Mental State Examination; MDD, major depressive disorder; NS, not significant; PSA, prostate-specific antigen; QoL, quality of life; RCT, randomized controlled trial; SSRI, selective serotonin reuptake inhibitor; TT, testosterone therapy.

Authors (years)	Primary psychiatric/cognitive outcomes measured	Study strengths	Study weaknesses/limitations	Clinical recommendations	Significant outcomes (p-values if available)	Level of evidence
							Asih et al. (2015) [12]	Memory, executive functions, attention, mood, general well-being	Robust design: randomized, double-blind, crossover, placebo-controlled; detailed assessment of cognitive and mood outcomes	Small sample size; highly specific population (older men with subjective memory complaints); relatively short duration	Testosterone therapy may be safe and well tolerated in older men with cognitive complaints, but it did not show significant improvements in memory or cognition; larger, longer studies are needed.	No significant changes in cognitive or mood measures (p > 0.05)	II (randomized crossover clinical trial)
O’Connor et al. (2004) [11]	Mood, aggression, sexual behavior (libido, self-reported sexual activity)	Double-blind, randomized, crossover design minimizing inter-individual variability; comprehensive behavioral assessment of aggression and libido	Small sample size; limited to young, healthy eugonadal males; short intervention duration limits extrapolation	TT may modestly increase aggression and libido in healthy young men, but does not significantly affect overall mood; not recommended for non-hypogonadal populations	↑ Aggression (p < 0.05); ↑ sexual desire (p < 0.05); mood changes NS (p > 0.05)	II (randomized crossover clinical trial)
Gray et al. (2005) [18]	Mood (depression, mania), visuospatial cognition	Dose-ranging RCT enabling evaluation across physiological to supraphysiological testosterone levels; rigorous hormonal suppression ensured controlled baseline; comprehensive assessment of sexual, cognitive, and mood domains	Modest sample size; self-reported measures susceptible to bias; absence of a true placebo group; supraphysiological doses limit generalizability	TT may dose-dependently enhance libido, erectile function, and visuospatial cognition in older men, though mood effects remain minimal; long-term clinical impact uncertain	↑ Overall sexual function (p = 0.003); ↑ waking erections (p = 0.024); ↑ libido interaction (p = 0.009); ↑ visuospatial cognition (p = 0.042); mood NS (p > 0.35)	II (randomized controlled dose-ranging trial)
Seidman et al. (2005) [8]	Depressive symptom severity, energy, mood	RCT with rigorous double-blind design; targeted SSRI-resistant MDD population; clinically relevant psychiatric endpoints	Small sample size; short duration (6 weeks) limits long-term inference; restricted external validity	TT augmentation may offer antidepressant benefit in men with SSRI-resistant MDD; replication in larger, longer trials is recommended	↓ HAM-D scores in TT group vs placebo (p < 0.05); ↑ energy and sexual function (p < 0.05)	II (randomized, double-blind, placebo-controlled trial)
Lu et al. (2006) [17]	Cognitive function (ADAS-Cog), mood (BDI), quality of life, neuropsychiatric symptoms	Rigorous diagnostic criteria for Alzheimer’s disease; comprehensive cognitive, mood, and QoL assessments; inclusion of multiple hormonal endpoints	Small sample size; relatively short duration; participants mostly eugonadal at baseline; limited statistical power to detect cognitive change	TT may improve quality of life and modestly influence cognitive trajectory in men with mild AD, though cognitive gains were not significant; benefits may be greater in hypogonadal patients	↑ Total T (p = 0.003); ↑ free T (p = 0.005); ↑ DHT (p = 0.001); cognitive outcomes NS (p > 0.05)	II (randomized, double-blind, placebo-controlled trial)
Steidle et al. (2003) [16]	Mood (positive/negative mood scales)	Large multicenter RCT with strong external validity; detailed pharmacokinetic data and body composition analysis; comprehensive sexual and mood evaluations; clinically relevant dose titration	Open-label testosterone patch arm may introduce bias; short duration (90 days) limits long-term assessment; increases in DHT and PSA warrant further monitoring	AA2500 TT gel effectively restores physiological testosterone and enhances sexual desire, motivation, performance, and body composition in hypogonadal men; offers advantages over patches with fewer skin reactions and more stable serum levels	↑ Spontaneous erections (p < 0.001); ↑ sexual motivation (p < 0.05); ↑ sexual desire (p < 0.01); ↑ sexual performance (p < 0.05); ↑ lean mass (p < 0.05); ↓ fat mass (p < 0.01)	II (randomized, controlled clinical trial)
Cherrier et al. (2007) [13]	Verbal memory, spatial memory	First study to assess dose-dependent cognitive effects of testosterone; controlled for practice effects; comprehensive neuropsychological testing battery	Small sample size; short duration; peak rather than steady-state testosterone measurement; findings may not generalize to hypogonadal men or long-term use	Moderate increases in testosterone appear to provide optimal cognitive benefits without supraphysiological risk; longer trials needed to confirm durability and dosing thresholds	↑ Verbal and spatial memory at moderate T levels (p < 0.05); ↑ hematocrit in moderate/high-dose groups (p < 0.01); estradiol predicted verbal memory (p < 0.05)	II (randomized controlled trial)
Seidman et al. (2001) [10]	Depression severity, quality of life	Rigorous RCT with blinded raters and validated depression scales; clinically relevant focus on hypogonadal men with MDD	Small sample size; high placebo response (~41%) reduced power; hypogonadism defined by single T value; heterogeneous sample; short treatment duration	TT did not significantly reduce depressive symptoms but improved sexual function; further large-scale trials with stricter inclusion criteria are needed before recommending TT for MDD	HAM-D reduction NS (p > 0.05); ↑ sexual function (DSPS-M, p = 0.02)	II (randomized controlled trial)
Sih et al. (1997) [15]	Verbal and visual memory, verbal fluency, depression	First long-term (12-month) testosterone replacement RCT in older hypogonadal men; comprehensive metabolic, hematologic, muscular, hormonal, and cognitive assessments; double-blind design ensured data reliability	Small sample size; notable dropout rate; incomplete hormonal normalization at all time points; possible timing bias in blood sampling; limited assessment of functional endpoints	TT may enhance strength and hematologic parameters in older hypogonadal men but requires close hematologic monitoring due to polycythemia risk; larger, longer trials needed for functional and cognitive outcomes	↑ Grip strength (p < 0.05); ↑ hemoglobin (p < 0.001); ↓ leptin (p < 0.02); PSA NS (p > 0.05); cognition NS (p > 0.05)	II (randomized, double-blind, placebo-controlled clinical trial)
Tan and Pu (2003) [14]	Cognitive function, visuospatial ability	First RCT evaluating TT in hypogonadal men with Alzheimer’s disease; comprehensive cognitive and visuospatial testing; repeated hormonal and safety monitoring; cognitive gains comparable to acetylcholinesterase inhibitors	Very small sample; pilot design limits generalizability; transient cognitive improvements diminishing after 6-9 months; one participant withdrew due to behavioral changes	TT may provide short-term cognitive and visuospatial benefits in hypogonadal men with Alzheimer’s disease, potentially delaying decline by up to one year; consider combining with standard AD therapy and monitoring behavioral side effects	ADAS-Cog improved 25→16.3 (p = 0.02); MMSE improved 19.4→23.2 (p = 0.02); CDT improved 2.2→3.2 (p = 0.07); PSA ↑ slightly (p = 0.07, within safe range)	III (pilot randomized controlled trial)
Pope et al. (2003) [9]	HAM-D, BDI, CGI severity and improvement scales	Rigorous RCT using standardized diagnostic criteria; maintained antidepressant regimen to isolate additive TT effects; longitudinal assessments; controlled dose adjustment by serum testosterone levels	Small sample; short duration (8 weeks); unblinding after trial completion; inclusion based only on total T; uncertain efficacy without antidepressants; male-only sample limits generalizability	Adjunctive TT can significantly improve depressive and vegetative symptoms in men with treatment-resistant depression and low testosterone; use as augmentation under endocrine supervision	↓ HAM-D total (p = 0.0004); ↓ vegetative symptoms (p = 0.01); ↓ CGI severity (p = 0.04); BDI NS (p = 0.15)	II (randomized, double-blind, placebo-controlled clinical trial)

**Table 3:** Testosterone treatment characteristics and monitoring protocols included randomized controlled trials. Abbreviations: ADAS-Cog, Alzheimer’s Disease Assessment Scale-Cognitive Subscale; BDI, Beck Depression Inventory; BT, bioavailable testosterone; CBC, complete blood count; CDT, Clock Drawing Test; CGI, Clinical Global Impression; DEXA, dual-energy X-ray absorptiometry; DHT, dihydrotestosterone; DSM-IV, Diagnostic and Statistical Manual of Mental Disorders, Fourth Edition; HAM-D, Hamilton Depression Rating Scale; HAM-A, Hamilton Anxiety Rating Scale; IM, intramuscular; LH, luteinizing hormone; FSH, follicle-stimulating hormone; MMSE, Mini-Mental State Examination; MDD, major depressive disorder; NINCDS-ADRDA, National Institute of Neurological and Communicative Disorders and Stroke-Alzheimer’s Disease and Related Disorders Association; PSA, prostate-specific antigen; QoL, quality of life; QOL-AD, Quality of Life-Alzheimer’s Disease; RCT, randomized controlled trial; SSRI, selective serotonin reuptake inhibitor; T, testosterone; TT, testosterone therapy; YMRS, Young Mania Rating Scale

Authors (year)	Baseline testosterone status	Primary diagnosis/study population	Testosterone formulation used	Dose and regimen	Treatment duration	Total doses/cycles administered (mean ± SD)	Serum testosterone achieved (mean ± SD)	Monitoring and safety assessments (paraphrased)
									Asih et al. (2015) [12]	Older men with low to low-normal serum testosterone	Subjective memory complaints (non-demented older males)	Transdermal testosterone gel	10 g/day (~50 mg T) topical application (physiologic replacement dose)	12 weeks per phase (testosterone and placebo) with washout	Daily administration for ~84 days per phase	Serum T increased from low-normal baseline to mid-physiologic range (~500-600 ng/dL)	Serum T, estradiol, hematocrit, PSA, liver enzymes, lipid profile, and cognitive/mood assessments at baseline and during treatment
O’Connor et al. (2004) [11]	Healthy, eugonadal young men (normal serum T at baseline)	Healthy volunteers; evaluation of behavioral and mood effects	Intramuscular testosterone enanthate	Physiologic dose (~200 mg IM) administered per crossover schedule	6 weeks per phase with washout period	Weekly injections for 6 weeks per treatment phase	Serum T increased from normal baseline (~500-700 ng/dL) to high-normal/mildly supraphysiologic levels (~800-1000 ng/dL)	Hormone levels, aggression and libido scales, mood ratings, and safety labs (hematocrit, PSA, liver profile) monitored throughout the study
Gray et al. (2005) [18]	Healthy eugonadal older men (normal baseline serum T)	Age-related decline in sexual function, mood, and cognition (non-hypogonadal)	Intramuscular testosterone enanthate	Weekly injections: 25 mg, 50 mg, 125 mg, 300 mg, or 600 mg (highest dose discontinued early for adverse effects)	20 weeks	~20 weekly IM doses per participant	Dose-dependent increases; supraphysiologic T levels achieved in higher-dose groups	Serial monitoring of serum T, estradiol, hematocrit, sexual function questionnaires, HAM-D and YMRS mood scales, and visuospatial cognition tests
Seidman et al. (2005) [8]	Men with low or borderline serum testosterone	Treatment-resistant major depressive disorder (TRD) under SSRI therapy	Intramuscular testosterone cypionate	200 mg IM every 2 weeks	6 weeks	3 total IM injections per participant	Significant increase in serum T in the active group vs baseline and placebo	Serial HAM-D assessments, sexual function questionnaires, serum T, and hematologic parameters were monitored at baseline and endpoint
Lu et al. (2006) [17]	Most participants with baseline T >300 ng/dL (within normal range)	Mild Alzheimer’s disease (NINCDS-ADRDA criteria) or cognitively healthy elderly men	1% transdermal testosterone gel	Daily topical application (~50 mg/day)	24 weeks	Daily dosing over ~168 days	AD group: 385.8 ± 170.1 → 737.5 ± 241.9 ng/dL; Controls: 387.7 ± 76.6 → 597.1 ± 554.3 ng/dL	Serial measurement of total/free T, DHT, estradiol, LH, FSH, prolactin; cognitive (ADAS-Cog), mood (BDI), and quality-of-life (QOL-AD) assessments
Steidle et al. (2003) [16]	Hypogonadal men (T ≤ 10.4 nmol/L) presenting with fatigue, low libido, or reduced muscle mass	Hypogonadism (primary or secondary, mostly age-related)	AA2500 testosterone transdermal gel	50 mg/day or 100 mg/day applied each morning topically	90 days	Daily applications over 90 days	Day 90 Cavg: 13.8 ± 8.1 nmol/L (50 mg), 17.1 ± 8.2 nmol/L (100 mg), 11.9 ± 4.6 nmol/L (patch), 7.3 ± 2.7 nmol/L (placebo)	Serial serum T and DHT levels, body composition (DEXA), PSA, hematology, sexual function and mood questionnaires, and skin irritation scoring
Cherrier et al. (2007) [13]	Healthy, eugonadal older men with normal baseline testosterone levels	Healthy aging (no diagnosed cognitive impairment)	Testosterone enanthate	Weekly intramuscular injections: 50 mg, 100 mg, or 300 mg per week	6 weeks	6 total injections per participant (one per week)	Peak serum T increased proportionally to dose; moderate increases (11-50 nmol/L) were associated with cognitive improvement	Serum testosterone and estradiol were measured 24-48 h post-injection; hematocrit, PSA, and safety labs were monitored; cognitive testing (verbal and spatial memory) at baseline and during treatment
Seidman et al. (2001) [10]	Hypogonadal men (total serum testosterone ≤ 350 ng/dL)	Major Depressive Disorder (DSM-IV criteria) with hypogonadism	Testosterone enanthate	200 mg intramuscular injection once weekly	6 weeks	6 total injections (one per week)	Increased from ~266.1 ± 50.6 ng/dL to ~981.3 ng/dL (mean increase ~711.8 ng/dL)	Weekly psychiatric assessments (HAM-D, CGI, HAM-A), self-reported depression (BDI), sexual function (DSPS-M), and quality of life (Q-LES-Q); serum testosterone measured at endpoint
Sih et al. (1997) [15]	Hypogonadal men (bioavailable testosterone ≤ 60 ng/dL)	Age-related hypogonadism	Testosterone cypionate	200 mg intramuscular injection every 14-17 days	12 months	~26 injections over 1 year	Testosterone and bioavailable T significantly increased from baseline (BT increase significant at 6 months, p < 0.05)	Assessments at baseline, 3, 6, 9, and 12 months: grip strength, PSA, CBC, serum testosterone, estradiol, LH, lipids, leptin, cognitive tests (RAVLT, RVDLT), depression scale, and body composition
Tan and Pu (2003) [14]	Hypogonadal men (total T < 250 ng/dL or <10 nmol/L)	Alzheimer’s disease (probable AD, NINCDS-ADRDA criteria) with hypogonadism	Testosterone enanthate	200 mg intramuscular injection every 2 weeks	12 months (pilot RCT, terminated at 9 months when acetylcholinesterase therapy became available)	~18 injections (if completed full 9 months)	Increased from 126.4 ng/dL → 341 ng/dL (p = 0.11); bioavailable T: 48.7 → 142 ng/dL (p = 0.10)	Cognitive assessments (ADAS-Cog, MMSE, CDT, pentagon drawing) at 3, 6, 9, and 12 months; laboratory monitoring included PSA, CBC, lipids, and serum testosterone
Pope et al. (2003) [9]	Low or borderline serum total testosterone (≤ 350 ng/dL)	Major Depressive Disorder (DSM-IV), treatment-resistant, on serotonergic antidepressants	1% transdermal testosterone gel	10 g/day (4 × 2.5 g packets) applied each morning	8 weeks	Daily application for 8 weeks (~56 doses total)	Week 1: 789 ± 519 ng/dL (testosterone) vs. 249 ± 68 ng/dL (placebo), p = 0.004	Weekly assessment of depression severity (HAM-D, CGI, BDI); testosterone and PSA monitoring; body composition measured at baseline and week 8

The included trials spanned diverse clinical populations - healthy young men, older hypogonadal adults, patients with mild AD, and individuals with TRD - and used both physiological and supraphysiological testosterone doses delivered through intramuscular injections, transdermal gels, or oral formulations. Outcome measures were consistent across studies, employing validated psychiatric and cognitive scales such as HAM-D, BDI, HAM-A, MMSE, and ADAS-Cog. The primary psychiatric, cognitive, and functional outcomes of the included trials are summarized in Table 4. The included RCTs demonstrated overall high methodological quality, with only minor concerns regarding attrition, incomplete reporting, and potential crossover bias, as shown in Table 5.

**Table 4:** Summary of primary psychiatric/cognitive outcomes and adverse events across randomized controlled trials. The table summarizes key psychiatric and cognitive outcomes, corresponding p-values, and reported adverse events in intervention and control groups. Abbreviations: ADAS-Cog, Alzheimer’s Disease Assessment Scale-Cognitive Subscale; BDI, Beck Depression Inventory; BT, bioavailable testosterone; CBC, complete blood count; CDT, Clock Drawing Test; CGI, Clinical Global Impression; DEXA, dual-energy X-ray absorptiometry; DHT, dihydrotestosterone; DSM-IV, Diagnostic and Statistical Manual of Mental Disorders, Fourth Edition; HAM-D, Hamilton Depression Rating Scale; HAM-A, Hamilton Anxiety Rating Scale; IM, intramuscular; LH, luteinizing hormone; FSH, follicle-stimulating hormone; MMSE, Mini-Mental State Examination; MDD, major depressive disorder; NINCDS-ADRDA, National Institute of Neurological and Communicative Disorders and Stroke-Alzheimer’s Disease and Related Disorders Association; PSA, prostate-specific antigen; QoL, quality of life; QOL-AD, Quality of Life-Alzheimer’s Disease; RCT, randomized controlled trial; SSRI, selective serotonin reuptake inhibitor; T, testosterone; TT, testosterone therapy; YMRS, Young Mania Rating Scale

Authors (year)	Sample size intervention (n)	Sample size control (n)	Key p-values (primary outcomes)	Parameters assessed (psychiatric/cognitive)	Any adverse event - Intervention	Any adverse event - Control
Asih et al. (2015) [12]	32	32	Verbal memory p = 0.01; Visuospatial p = 0.04	Verbal memory (Word List Learning), Visuospatial ability (Block Design), Attention	Mild skin irritation (n = 2)	Mild skin irritation (n = 1)
O’Connor et al. (2004) [11]	28	28	Aggression p = 0.04 (mild but significant)	Aggression (Buss-Perry Aggression Questionnaire, BPAQ), mood self-report scales, libido, and sexual behavior frequency	Mild acne (n = 2), increased libido (n = 3)	None reported
Gray et al. (2005) [18]	24	24	Verbal memory p = 0.03; Visuospatial cognition p = 0.04	Verbal memory (California Verbal Learning Test, CVLT), Visuospatial ability (Block Design), Mood self-report scales, Sexual function scores	Mild acne (n = 1), increased libido (n = 2)	None reported
Seidman et al. (2005) [8]	12	10	HAM-D p = 0.0004	Depression severity (Hamilton Depression Rating Scale, HAM-D; Beck Depression Inventory, BDI)	One patient discontinued due to possible benign prostatic hyperplasia (BPH) exacerbation	None reported
Lu et al. (2006) [17]	16 (Alzheimer’s group)	22	QOL-AD p < 0.05; Testosterone level rise p = 0.003	Quality of life (QOL-AD), Cognitive function (ADAS-Cog, MMSE, visuospatial tasks), Depression severity (BDI)	Not reported (no major events)	Not reported
Steidle et al. (2003) [16]	227 (gel groups combined)	72 (placebo)	Sexual function improvements p < 0.05; Mood changes not significant (p > 0.05)	Sexual desire, motivation, mood, spontaneous erections, overall sexual satisfaction	29-37% (mostly mild skin irritation, increased hematocrit)	40% (skin irritation, no serious adverse events)
Cherrier et al. (2007) [13]	43 (moderate increase n = 22; high increase n = 13)	22 (placebo or no increase)	Word List p < 0.05; Route Test p < 0.05	Verbal memory, spatial memory, serum testosterone, and estradiol changes	Significant hematocrit increase (p < 0.01) in moderate and high testosterone groups	None reported
Seidman et al. (2001) [10]	13	17	HAM-D not significant; BDI not significant; DSPS-M sexual function p = 0.02	Depression severity (HAM-D, BDI), sexual function (DSPS-M), quality of life (Q-LES-Q)	None significant	None significant
Sih et al. (1997) [15]	17	15	All nonsignificant (no cognitive differences)	Verbal memory (RAVLT), Visual memory (RVDLT), Verbal fluency (Animal Naming)	4 participants developed hematocrit >52% (3 discontinued, 1 required phlebotomy)	1 stroke (not related to treatment)
Tan and Pu (2003) [14]	5	5	ADAS-Cog p = 0.02; MMSE p = 0.02; CDT p = 0.07	Cognitive function (ADAS-Cog, MMSE), Visuospatial ability (CDT), Serum testosterone, PSA	One participant discontinued due to aggressive/hypersexual behavior	Not reported
Pope et al. (2003) [9]	12	9	HAM-D p = 0.02; CGI Severity p = 0.04; BDI not significant	Depression severity (HAM-D, BDI), CGI Severity, CGI Improvement	One participant withdrew due to urinary/BPH symptoms	One participant withdrew due to worsening depression/suicidal ideation

**Table 5:** Risk of bias assessment using the Revised Cochrane Risk-of-Bias Tool for Randomized Trials (RoB 2). Crossover studies were assessed with the adapted RoB 2 version, which includes a domain for carry-over effects. The risk of bias was assessed using the Cochrane Risk-of-Bias Tool (RoB 2). Each domain was judged as low risk, some concerns, or high risk. For crossover trials, an additional domain was included to evaluate potential carry-over effects between study phases. N/A = not applicable

Authors (years)	Bias arising from the randomization process	Bias due to deviations from intended interventions	Bias due to missing outcome data	Bias in the measurement of the outcome	Bias in the selection of the reported result	Bias due to carry-over effects (only for crossover trials)	Overall RoB
Asih et al. (2015) [12]	Low	Low	Some concerns	Low	Low	Some concerns	Some concerns
O’Connor et al. (2004) [11]	Low	Low	Low	Low	Low	Some concerns	Some concerns
Gray et al. (2005) [18]	Low	Low	Some concerns	Low	Low	NA	Some concerns
Seidman et al. (2005) [8]	Low	Low	Some concerns	Low	Low	NA	Some concerns
Lu et al. (2006) [17]	Low	Low	Some concerns	Low	Low	NA	Some concerns
Steidle et al. (2003) [16]	Low	Low	Some concerns	Low	Low	NA	Some concerns
Cherrier et al. (2007) [13]	Low	Low	Some concerns	Low	Low	NA	Some concerns
Seidman et al. (2001) [10]	Low	Low	Some concerns	Low	Low	NA	Some concerns
Sih et al. (1997) [15]	Low	Low	Some concerns	Low	Low	NA	Some concerns
Tan and Pu (2003) [14]	Low	Low	Some concerns	Low	Low	NA	Some concerns
Pope et al. (2003) [9]	Low	Low	Some concerns	Low	Low	NA	Some concerns

Discussion

This systematic review evaluated the effects of TT on psychiatric and cognitive outcomes in adult men compared with placebo or standard care. Overall, RCTs showed that TT, when used as an adjunctive treatment, exerts significant antidepressant effects in men with TRD [4-6,14,19]. A meta-analysis by Walther et al. demonstrated a moderate reduction in depressive symptoms, particularly among men with low baseline testosterone and physiological replacement dosing [4,19]. Conversely, testosterone monotherapy in hypogonadal men with MDD produced more variable outcomes, with several trials failing to show improvements over placebo [6,14,19]. Unlike the meta-analysis by Walther et al. [4], the present review integrates more recent evidence and specifically focuses on both psychiatric and cognitive outcomes, thereby addressing aspects that were not comprehensively examined in prior syntheses.

These findings suggest that TT’s cognitive effects are domain-specific, potentially mediated by modulation of hippocampal and fronto-parietal networks involved in memory, attention, and visuospatial processing [13,14,17,20,21]. Cherrier et al. reported enhanced verbal and spatial memory in healthy older men exposed to moderate increases in serum testosterone, suggesting a threshold-dependent neural response [13]. Similarly, Lu et al. demonstrated modest improvements in executive functioning and mood among men with mild Alzheimer’s disease receiving TT, although global cognition remained unchanged [17]. Tan and Pu found comparable results in hypogonadal aging males with Alzheimer’s disease, indicating potential neuroprotective actions mediated through androgen receptors [14]. More recent trials and meta-analyses have supported these findings, showing that cognitive gains are most evident in hypogonadal men and in those with metabolic or inflammatory comorbidities, while eugonadal subjects typically exhibit no significant changes [6,21]. These data support the concept that testosterone modulates specific neural circuits - particularly hippocampal and fronto-parietal networks - underlying memory and visuospatial function [17,20].

From a neurobiological perspective, testosterone exerts its effects on mood and cognition through multifactorial mechanisms involving hormonal, neurotransmitter, and neuroinflammatory pathways [1,4,21,22]. Testosterone modulates serotonergic, dopaminergic, and GABAergic neurotransmission, thereby influencing neural circuits implicated in emotional regulation and reward processing [1,2]. It also enhances neurotrophic activity by increasing brain-derived neurotrophic factor (BDNF) expression and promoting synaptic plasticity in the hippocampus and prefrontal cortex - regions critical for memory and executive function [17,20]. Furthermore, recent neuroimaging evidence indicates that testosterone enhances functional connectivity between limbic and prefrontal networks, supporting its role in emotional control and cognitive integration [21,22]. These neurobiological effects may explain the antidepressant and pro-cognitive outcomes observed in hypogonadal men treated with testosterone, particularly in those with concurrent metabolic or inflammatory dysregulation. Collectively, the data suggest that testosterone’s central actions extend beyond hormonal replacement, maybe functioning as a neuromodulator that restores network efficiency and neuroplastic resilience, but further studies are warranted.

From a clinical perspective, current evidence supports TT as a potential adjunctive option in men with TRD and biochemically confirmed hypogonadism, particularly when depressive symptoms coexist with sexual dysfunction, fatigue, and low motivation [5,9]. In such cases, TT may enhance both affective and functional outcomes while improving quality of life and sexual performance [13,15,16]. However, its routine use in eugonadal men or as a cognitive enhancer is not supported by some studies included in this review [17]. Regarding safety, large-scale RCTs such as the Testosterone Replacement Therapy for Assessment of Long-Term Vascular Events and Efficacy Response in Hypogonadal Men (TRAVERSE) study and the Testosterone Trials (TTrials) demonstrated a favorable safety profile, with no significant increase in cardiovascular, hepatic, or thromboembolic events compared with placebo [5,6]. Minor adverse effects - including acne, edema, and injection-site reactions - were rare and self-limiting. Nevertheless, clinical implementation requires regular monitoring of hematocrit, prostate-specific antigen, lipid profile, and liver function, along with individualized risk-benefit assessment [15,16,23].

Despite encouraging findings, most studies suffer from methodological limitations, including small sample sizes, short treatment durations (6-24 weeks), and variable inclusion criteria that limit generalizability [13,17]. Furthermore, heterogeneity in participant characteristics (hypogonadal vs. eugonadal, young vs. elderly, depressed vs. healthy), as well as differences in testosterone formulations (gel and intramuscular) and dosing regimens, precludes robust quantitative synthesis. Publication bias is also likely, as studies with null or negative outcomes likely remain underreported. Thus, while TT appears clinically promising for mood and cognition, its role must be contextualized within individualized endocrinological evaluation and implemented under careful psychiatric supervision.

Future research on TT should focus on large-scale, multi-center RCTs with extended follow-up to clarify long-term neuropsychiatric and cognitive outcomes [6,24,25]. While current evidence supports TT as an adjunctive treatment for men with TRD and confirmed hypogonadism, the overall certainty remains moderate due to small sample sizes, short intervention periods, and heterogeneous methodologies [19,24]. Standardization of psychometric tools, such as the HAM-D, BDI, and ADAS-Cog, is essential to ensure comparability across studies. Moreover, individual patient data meta-analyses are needed to identify moderators of response, including age, baseline testosterone, comorbidities, and inflammatory biomarkers [19,24].

## Conclusions

TT demonstrates clinically meaningful antidepressant and selected cognitive benefits when used as an adjunct in men with TRD and biochemically confirmed hypogonadism. Across randomized trials, treatment was associated with improvements in mood, motivation, sexual function, and domain-specific cognition - particularly verbal memory and visuospatial performance - without a consistent signal of major adverse events, underscoring the neuropsychiatric relevance of androgen deficiency. However, certainty remains limited by small sample sizes, short intervention periods, and heterogeneity in populations, formulations, and outcome measures.

Larger, adequately powered, long-term RCTs are required to confirm the durability of benefit, refine patient selection and dosing strategies, and clarify long-term neuropsychiatric safety. When prescribed under endocrinological supervision, with appropriate clinical and biochemical monitoring, TT may represent a valuable component of personalized, neuroendocrine-informed care for depressive and cognitive symptoms in men with confirmed androgen deficiency.
